# A colonic splenic flexure tumour presenting as an empyema thoracis: a case report

**DOI:** 10.1186/1752-1947-3-9

**Published:** 2009-01-13

**Authors:** K Murphy, M Chaudry, HP Redmond

**Affiliations:** 1 Department of Surgery, Cork University Hospital, Wilton, Cork, Ireland

## Abstract

**Introduction:**

The case report describes the rare presentation of a 79-year-old patient with a locally perforated splenic flexure tumour of the colon presenting with an apparent empyema thoracis in the absence of abdominal signs or symptoms.

**Case presentation:**

Initial presentation was with a non-productive cough, anorexia and general malaise. An admission chest X-ray and subsequent computed tomographic image of the thorax showed a loculated pleural effusion consistent with an empyema. The computed tomography also showed a thickened splenic flexure. Thoracotomy was performed and a defect in the diaphragm was revealed after the abscess had been evacuated. A laparotomy was carried out at which point a tumour of the splenic flexure of the colon was found to be invading the spleen and locally perforated with subsequent collection in communication with the thorax. The tumour and spleen were resected and a transverse end colostomy was fashioned.

**Conclusion:**

One must consider the diagnosis of pathology inferior to the diaphragm when an apparent empyema thoracis is encountered even in the absence of clinical signs or symptoms.

## Introduction

The case describes the rare presentation of a patient with a locally perforated splenic flexure tumour of the colon with an apparent empyema thoracis in the absence of abdominal signs or symptoms. This has been described once in the past by Teruuchi *et al. *[1].

## Case presentation

**Figure 1:**
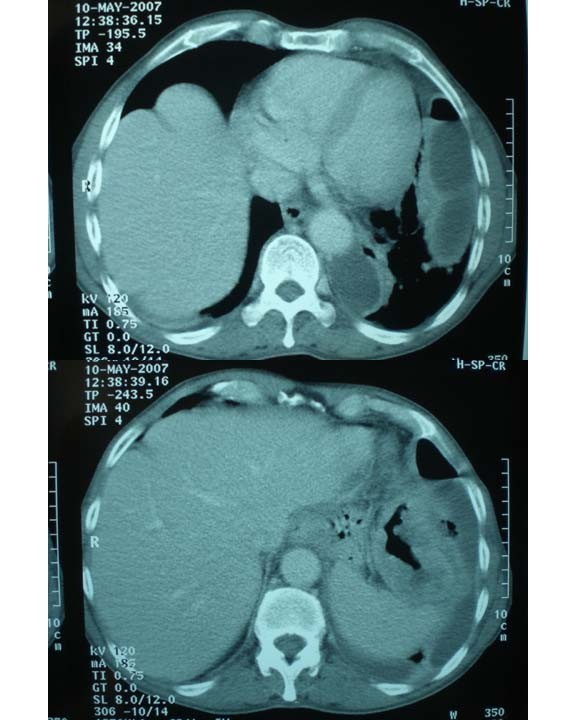
**Computed tomographic image of the loculated effusion and the thickened splenic flexure**.

This 79-year-old man presented to his local hospital in May 2007 with a 2-month history of a chronic non-productive cough, weight loss of 1.5 stone (9.5 kg) over 1 month, anorexia and general malaise. Other gastrointestinal or respiratory symptoms were absent. Examination was consistent with the finding of a left-sided pleural effusion and of note, abdominal exam was normal. An admission chest X-ray showed a left-sided pleural effusion with basal collapse. His inflammatory blood markers were also elevated (WCC 21.3, CRP 286). He was commenced on intravenous antibiotics but showed little improvement after 3 days of treatment, at which point a computed tomographic (CT) scan of the thorax and abdomen was performed. The CT showed a loculated pleural effusion consistent with an empyema plus thickening of the pleura and diaphragm as well as an abnormal thickened splenic flexure adjacent to the diaphragm (Figure 1). The patient was subsequently transferred for further management.

**Figure 2:**
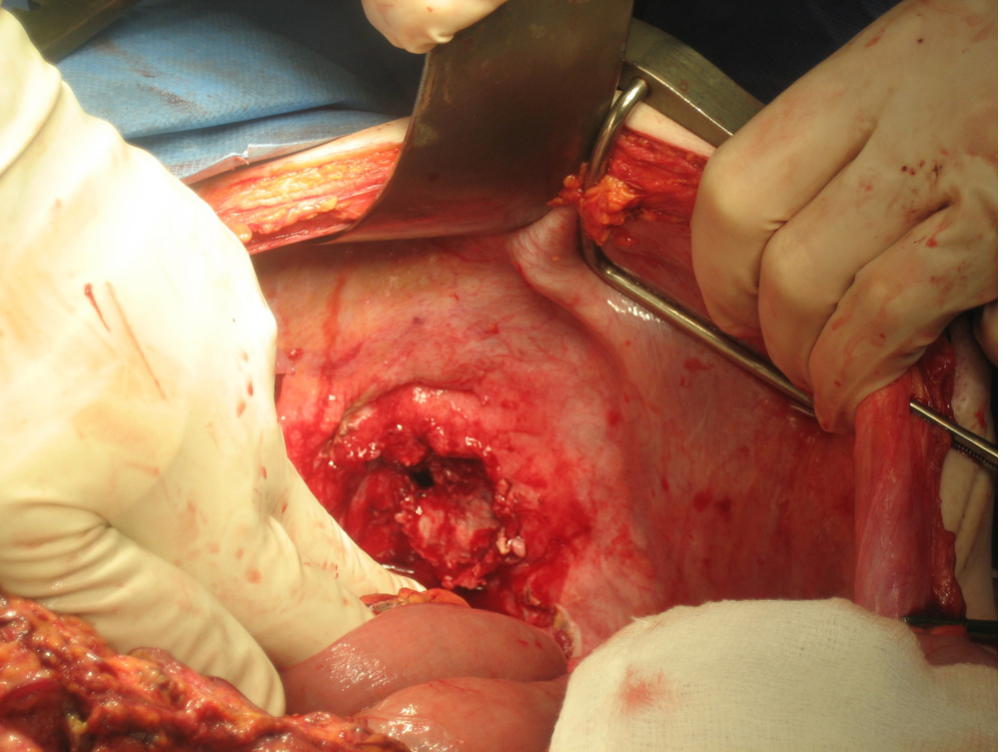
**Photograph of the diaphragmatic defect**.

**Figure 3:**
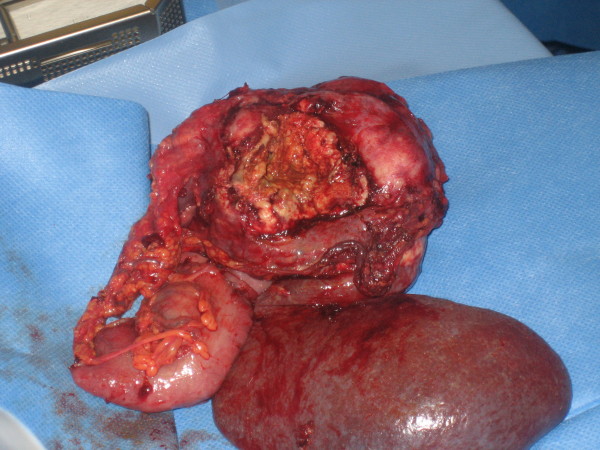
**Photographic evidence of the resected specimen with attached spleen**.

On the day of transfer, the patient was taken to theatre for pleural decortication. A posterolateral thoracotomy was performed and the abscess collection was evacuated. A small perforation of the diaphragm was also found and it was established that the abscess cavity was extending through the diaphragmatic defect (Figure 2). At this point a laparotomy was performed. It was found that the patient had a locally perforated splenic flexure tumour, which had formed a collection that communicated with the thorax and also invaded the spleen. The tumour, along with the adherent spleen was removed (Figure 3) and a transverse colostomy and distal mucus fistula were fashioned. An omental patch was used to close the defect in the diaphragm.

The patient made an uneventful postoperative recovery and was discharged from hospital 15 days after the operation having received the relevant vaccinations. Pathological assessment showed a Dukes B moderately differentiated adenocarcinoma with the pathological staging of T4 N0 M1. The label of M1 is derived from the fact that the thorax was breached. Adjuvant chemotherapy has been scheduled.

## Discussion

The case highlights the rare presentation of an apparent empyema thoracis secondary to abdominal pathology. Technically, it is not a true empyema given that it originated and communicated with the abdominal cavity, hence its labelling as an 'apparent' empyema. The only previous example of this pathology was described by Teruuchi *et al. *[1], and was the case of a 63-year-old man who presented with chest pain and fever and was noted to have a pleural effusion and a pneumothorax on chest X-ray. In contrast, our patient had neither chest pain nor a pneumothorax. What was also interesting in this instance is that the patient had an insidious onset of symptoms and had no abdominal symptoms or signs. Possible alternative approaches would have been to perform a contrast enema or colonoscopy pre-operatively to further evaluate the detected colonic anomaly or to carry out a thoracoscopy to assess the diaphragm even though one could argue that neither was clinically indicated. The use of video assisted thorascopic surgery (VATS) may have been advocated in some centres. Invariably, thoracotomy with washout as well as laparotomy with tumour resection would have been required, as was the procedure in our patient. In the future management of empyema thoracis, one needs to remember the differential diagnosis of diaphragmatic perforation that has been outlined in this patient, and that the clinical absence of classical symptoms and signs does not rule out the aforementioned pathology.

## Consent

Written informed consent was obtained from the patient for publication of this case report and any accompanying images. A copy of the written consent is available for review by the Editor-in-Chief of this journal.

## Competing interests

The authors declare that they have no competing interests.

## Authors' contributions

KM gathered the patient data, obtained consent from the patient and performed the literature review. MC was the major source of photographic images and was a major contributor in writing the manuscript. HPR was also a major contributor in writing the manuscript and was involved in the final histological assessment. All authors were intimately involved in the case, and read and approved the final manuscript.
